# Biomarkers in the pathogenesis and diagnosis of Crohn’s perianal fistulas: a systematic review

**DOI:** 10.1007/s10151-026-03376-8

**Published:** 2026-06-22

**Authors:** A. C. Silva, R. Magalhães, L. Lacerda, C. Robalo, P. Lago, M. D. Santos

**Affiliations:** 1 Serviço de Cirurgia Digestiva e Extradigestiva, Clínica de Cirurgia, Hospital de Santo António, Unidade Local de Saúde de Santo António, 4099-001 Porto, Portugal; 2 Serviço de Gastroenterologia, Hospital de Santo António, Unidade Local de Saúde de Santo António, 4099-001 Porto, Portugal; 3 Serviço de Genética Laboratorial, Clínica de Patologia e Genética - Centro de Genética Médica Jacinto de Magalhães, Unidade Local de Saúde de Santo António, 4099-028 Porto, Portugal; 4 Instituto de Ciências Biomédicas Abel Salazar, Universidade do Porto, Rua de Jorge Viterbo Ferreira, 228, 4050-313 Porto, Portugal; 5 Unidade Multidisciplinar de Investigação Biomédica, Instituto de Ciências Biomédicas Abel Salazar, Universidade do Porto, Rua Jorge Viterbo Ferreira 228, 4050-313 Porto, Portugal; 6 ITR - Laboratory for Integrative and Translational Research in Population Health, Rua das Taipas 135, 4050-600 Porto, Portugal

**Keywords:** Biomarkers, Crohn's disease, Perianal fistula, Diagnosis, Pathogenesis

## Abstract

**Background and aims:**

Perianal fistulas in Crohn’s disease (PFCD) continue to pose a therapeutic challenge owing to low rates of healing and remission. Identifying biomarkers involved in their pathogenesis and diagnosis is essential for personalising care and improving treatment outcomes. This systematic review aims to highlight PFCD biomarkers and provide insights for clinical practice.

**Methods:**

This systematic review, registered at PROSPERO (CRD42024575480) and conducted in accordance with PRISMA guidelines, includes English-language studies on PFCD biomarkers published through May 2025. Searches were performed in PubMed and Scopus. Study selection and risk-of-bias assessment were conducted independently by two reviewers, according to prespecified search and eligibility criteria.

**Results:**

Of 5242 records initially identified, 3700 were screened, and 33 studies were included (24 addressing pathogenesis and 9 diagnostic biomarkers). Variants in *OCTN1/2*, *NOD2, IRGM, TYK2*, and *VDR* genes influence fistula risk and severity in Crohn’s disease, particularly polymorphisms in *IRGM, NOD2*, and genes within the JAK–STAT signalling pathway. Altered microbiota and the presence of antibiotic resistance genes support a bacterial role in pathogenesis. Key pro-inflammatory cytokines (IL-1β, TNF-α, IL-6) and cellular populations (CD4⁺ T-cells, macrophages) drive inflammation, while epithelial-mesenchymal transition and extracellular matrix remodelling contribute to fistula persistence. Recent evidence suggests that PFCD arises from complex immune-stromal cell interactions, involving cellular senescence, epigenetic alterations, and population heterogeneity. Diagnostic advances include metabolic profiling and urinary Raman spectroscopy, alongside established biomarkers such as granulomas, faecal calprotectin, and NGAL.

**Conclusions:**

This review highlights several promising biomarkers involved in the pathogenesis and diagnosis of PFCD; however, heterogeneity across studies limits their immediate translation into clinical guidelines. Larger, well-designed studies are needed to validate these biomarkers and support personalised treatment strategies.

**Graphical abstract:**

**Figure:**
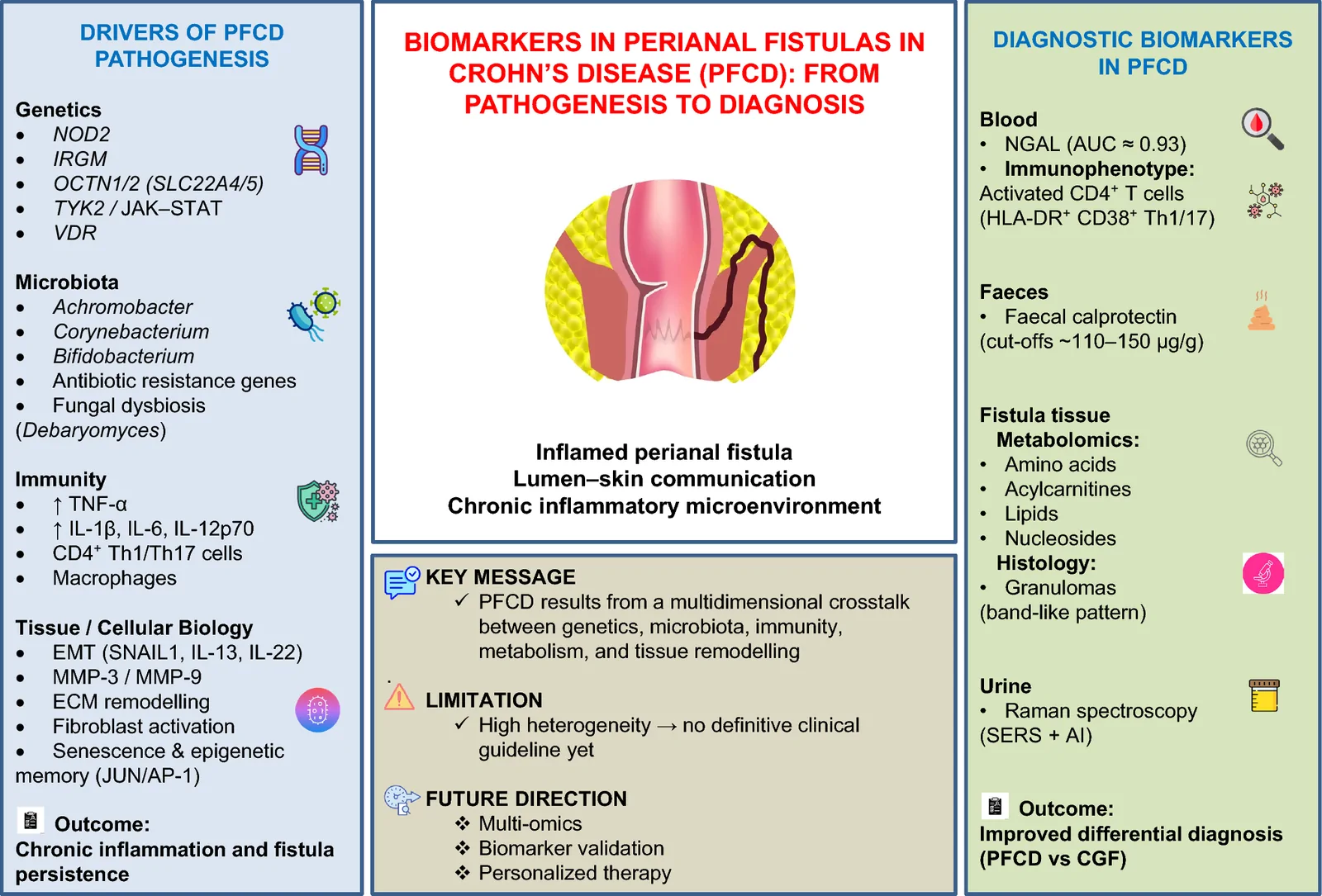

**Supplementary Information:**

The online version contains supplementary material available at https://doi.org/10.1007/s10151-026-03376-8.

## Introduction

Perianal fistulas (PF) are benign anorectal conditions that can significantly impair patients’ personal, social, and professional lives. Prompt and accurate diagnosis, together with appropriate treatment, are crucial for disease control and for improving patients’ quality of life [1]. However, PF recurrence rates following surgery are highly variable, ranging from 3 to 57%, and are influenced by factors such as anatomical classification, aetiology, and the therapeutic approach used [2].

While approximately 90% of PF cases are of cryptoglandular origin, other less common aetiologies include tuberculosis, trauma, hidradenitis suppurativa, human immunodeficiency virus (HIV) infection, sexually transmitted diseases, radiotherapy, and malignant diseases. Crohn’s disease (CD) represents a significant, though less frequent, cause. Recent studies indicate that PF and abscesses have a mean prevalence of 8–23 cases per 100,000 people, while perianal lesions associated with inflammatory bowel disease (IBD) affect approximately 4 cases per 100,000 individuals [3].

The pathogenesis of PF remains incompletely understood, and it is believed to involve distinct mechanisms in cryptoglandular fistulas (CGF) compared with perianal fistulas in Crohn’s disease (PFCD). The formation of these fistulas is a multifactorial process, potentially driven by anal gland and intestinal inflammation, epithelial-mesenchymal transition (EMT), and gut microbiota dysbiosis [4].

Cryptoglandular fistulas likely originate from infection of the perianal glands [5], which can progress to abscess and subsequent fistulisation. Pro-inflammatory cytokines drive tissue damage [6], while the microbiota appears to play a key role in sustaining the inflammatory process [7].

In PFCD, the secretion of pro-inflammatory cytokines such as tumour necrosis factor alpha (TNF-α) and transforming growth factor beta (TGF-β), combined with epithelial defects, can drive the EMT [8]. The microbiota may also contribute to both inflammation and EMT. Additionally, the activation of matrix metalloproteinases (MMPs) leads to inflammation, tissue damage, and, ultimately, fistula formation [9]. These complex interactions contribute to the low healing rate, high recurrence rate, and more aggressive behaviour of PFCD, which is often associated with a poorer overall prognosis in patients with CD [10].

The management of PFCD typically involves a combination of biological therapies and surgical procedures [1, 11]. This combined approach aims to control inflammation, preserve anal continence, and prevent disease recurrence. In contrast, the treatment of CGF is exclusively surgical or based on other local techniques, with no indication for systemic pharmacological therapy [12].

Distinguishing between these two types of PF is not always straightforward, especially when PF is the initial and sole manifestation of CD, which occurs in up to 10% of patients [13]. Therefore, accurate differential diagnosis is extremely important, as the therapeutic strategies differ markedly.

The primary objective of this review is to identify and synthesise the main biomarkers involved in the pathogenesis of PFCD. As a secondary objective, it aims to explore and identify biomarkers that may serve as diagnostic tools to distinguish CGF from PFCD, providing practical, patient-centred guidance for clinical decision-making.

## Methods

The systematic review was registered in the International Prospective Register of Systematic Reviews (PROSPERO, protocol ID: CRD42024575480), and the findings were reported following the Preferred Reporting Items for Systematic Reviews and Meta-Analyses (PRISMA) guidelines [14]. Deviations from the registered protocol were as follows: replacement of an author, modification of the review period, exclusion of the Embase database, and removal of age restrictions for participants.

### Search strategy

A systematic review was conducted of full-text articles published in English between January 2000 and May 2025. The PubMed and Scopus databases were searched. The search strategy was developed using a combination of Medical Subject Headings (MeSH) and free-text terms related to Crohn’s disease and perianal fistula. The full search strategies are available in the Supplementary Material. Reference lists of potentially relevant articles and previous literature reviews were also screened to identify additional studies.

### Study selection

Two reviewers (AS and CR) independently conducted database searches and removed duplicates using EndNote 21.

Titles, abstracts, and full texts of the articles were independently screened by the same two reviewers according to predefined eligibility criteria. In case of disagreement, a third senior reviewer (RM) independently assessed the full texts to reach a consensus.

### Eligibility criteria

Retrospective or prospective studies, case series, case-control studies, and cohort studies with a minimum sample size of 10 patients were included. Studies involving patients with PFCD diagnosed by clinical criteria, magnetic resonance imaging (MRI), or endoscopic ultrasound (EUS), and/or histology were considered. Studies assessing any biomarker associated with the pathogenesis or diagnosis of PF, as well as those comparing biomarker levels between patients with PFCD and healthy controls, between patients with PFCD and patients with CD without PF, or between patients with PFCD and patients with CGF, were also included.

We excluded studies involving patients with PF resulting from causes other than CD or cryptoglandular origin (including iatrogenic [postpartum or postsurgical], neoplastic, traumatic, HIV-related, or radiotherapy-induced fistulas), studies with a sample size of < 10 patients with fistulas, studies with unavailable full text, animal or in vitro studies, and studies focusing exclusively on clinical factors and treatment outcomes.

### Quality assessment

The methodological quality of the included studies was assessed by consensus between AS and CR using the Newcastle-Ottawa Scale (NOS) [15], applied to case-control and cohort studies, as well as its adapted version for cross-sectional studies, while retaining the classic nine-star scoring to ensure consistency. Based on the total score, studies were classified as high (7–9), moderate (4–6), or low quality (0–3), reflecting the risk of bias. Translational and experimental studies involving human tissue and in vitro models were evaluated using the adapted Quality Assessment Tool for In Vitro Studies (QUIN), considering cell characterization, use of appropriate controls, replication of experiments, validation of results (RNA-seq, qPCR, Western blot, immunofluorescence, spheroid models), statistical analysis, and consistency of conclusions.

## Results

**Fig. 1:**
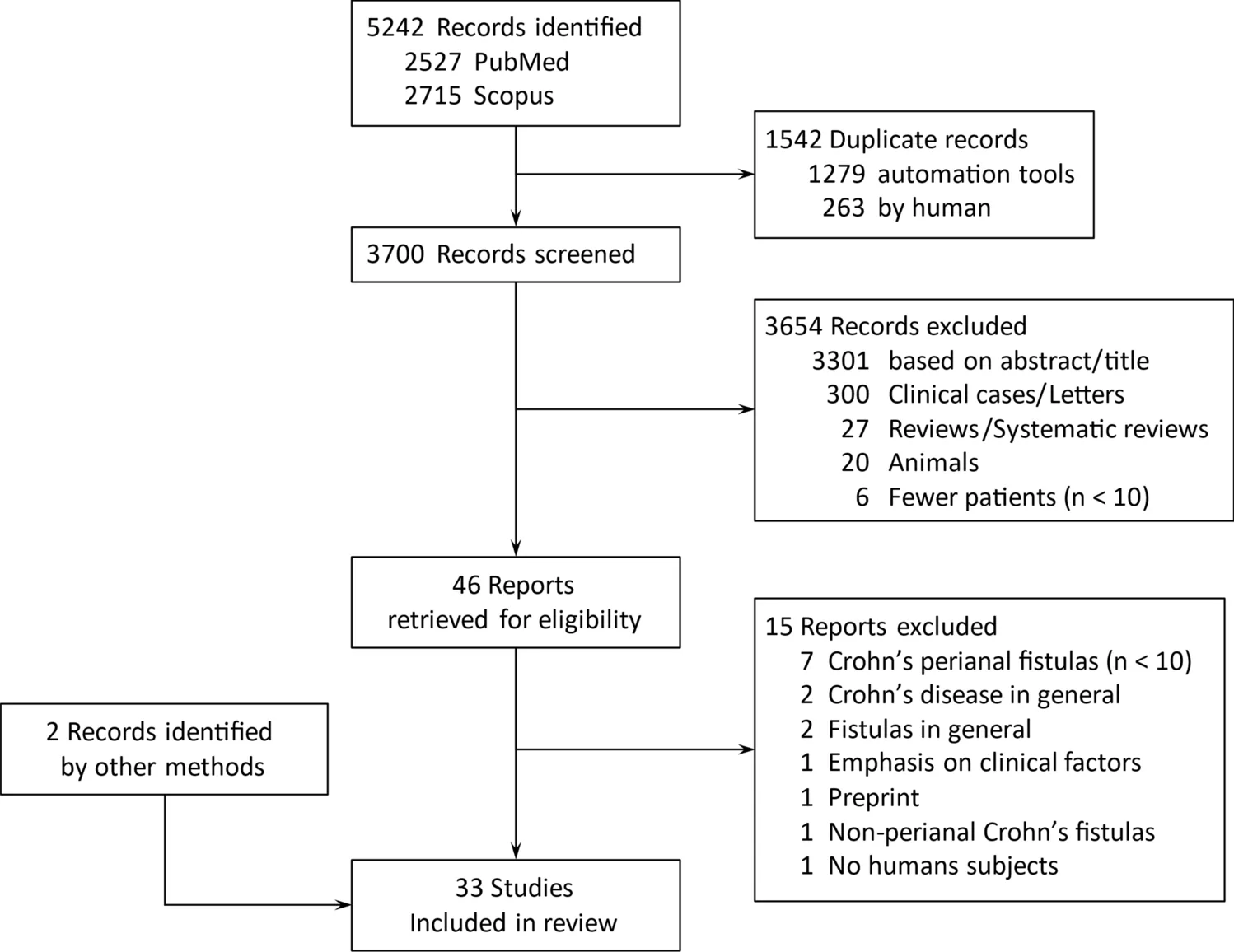
PRISMA flow diagram showing the study selection process

A total of 5242 records were identified through database searches: 2527 from PubMed and 2715 from Scopus. Following the removal of 1542 duplicate records, 3700 studies were screened by title and abstract. Forty-six full-text articles were assessed for eligibility, of which 31 met the inclusion criteria and were included in the final systematic review [16–48]. Screening of reference lists from the included studies identified two additional eligible studies. The review process is summarized in the PRISMA flow diagram (Fig. 1).

The included studies were conducted across diverse geographic regions, with 19 studies from European countries, 7 from the US, 6 from Asia, and 1 from New Zealand. A comprehensive description of the characteristics of these studies is provided in Tables 1 and 2.

**Table 1:** Main characteristics of the pathogenetic studies included in the analysis

Study (author, year, country)	Study type (period)	No. of participants	Aims	Biomarker	Outcomes
*Genetic profile*					
Vermeire et al*.* [17] 2005 Belgium	Case-control (NR)	981 IBD (203 PFCD) 305 HC	To study mutation prevalence, assess phenotypic expression, and perform conditional analyses to examine evidence for gene-gene interactions	OCTN (OCTN1 C1672T, OCTN2-207) *DLG5* (DLG5 G113A, DLG5_e26)	OCTN variants were specifically associated with perianal and penetrating CD
Palmieri et al. [18] 2006 Italy	Case-control (NR)	1510 total 444 CD (52 pCD) 455 UC 611 HC	To investigate these variants in CD and UC, their interaction with *CARD15* gene, and their correlation with clinical subphenotypes including PFCD	OCTN1 (*SLC22A4*): missense polymorphism 1672C**-**T OCTN2 (*SLC22A5*): promoter polymorphism-207G-C *CARD15:* variants R702W, G908R, and L1007fsinsC	TC haplotype is more frequent in CD patients with perianal involvement and stricturing/fistulising disease behaviour Organic cation transporter gene cluster variants may confer susceptibility to both CD and UC, and to PFCD
Jakubowska et al*.* [19] 2008 Poland	Cross-sectional (NR)	230 total 98 CD (25 PFCD) 132 HC	Assess polymorphisms in CD and their association to intestinal location and EIMs, including perianal disease	*CARD15/NOD2* G908R *DLG5* R30Q OCTN1 F503L	*CARD15/NOD2* G908R is associated with ileal disease but not with perianal disease
Henckaerts et al*.* [21] 2009 Germany	Cohort (1996–2009)	755 CD (48 PFCD) 367 HC	To examine the influence of recently discovered CD-associated susceptibility loci on changes in disease behaviour and to evaluate whether a genetic risk model for disease progression could be generated	*NOD2:* rs2066844, rs2066845, rs2066847 *L23R*: rs11209026 *IRGM:* rs13361189 *ATG16L1:* rs2241880 *CDKAL1:* rs6908425 *IL12B*: rs1363670 *SLC22A4/5:* Haplotype variants *TNFSF15:* rs4263839	The C allele of *CDKAL1* rs6908425 significantly increases the risk of developing PF (OR 8.86), whereas the presence of at least one *NOD2* mutation is associated with a lower risk (OR 0.56)
Latiano et al*.* [22] 2009 Italy	Case-control (2004–2005)	1754 total 823 CD (19 pCD) 353 UC 578 HC	To investigate whether these variants are associated with IBD in Italian patients with adult- and childhood-onset disease	*IRGM:* rs1000113, rs4958847	The *IRGM* rs4958847 polymorphism is associated with fistulising behaviour and PF in CD
Freire et al*.* [24] 2011 Portugal	Cohort (NR)	203 CD	To assess whether *CARD15* mutations are associated with the risk of developing PFCD and whether these mutations can predict the response of PF to antibiotic treatment	*CARD15/NOD2* mutations: R702W (SNP8), G908R (SNP12), and 3020insC (SNP13)	In CD, *CARD15* mutations are not associated with to the risk or timing of PF development Patients with PF carrying *CARD15* mutations showed poorer response to antibiotics (metronidazole ± ciprofloxacin)
Connelly et al*.* [25] 2012 USA	Cohort (1998–2011)	116 ileocolic CD (35 septic anal disease: abscesses/fistulas)	To identify SNPs associated with anal disease in general, septic anal disease specifically and the response to anti-TNF treatment	*TAGAP*: rs212388	SNP rs212388 showed a strong association with the presence and severity of anal disease in ileocolic CD Mutations in *TAGAP* may predict a milder form and course of pCD
Eglinton et al*.* [26] 2012 New Zealand	Case-control (2003–2005)	507 CD (137 pCD)	To identify the environmental, clinical, and genetic risk factors for pCD	*NOD2/CARD15*: R702W, G908R, 1007 fs *NOD2*: rs72796353 *IL2_21* *IL23R* *ATG16L1* *NCF4*: rs4821544 *IGRM* *TNF-α* *IBD5* (*OCTN1/2)* *NALP3* *CARD8*	Only *NCF4* showed a significant association with pCD *IBD5* (*OCTN1/2*); *TNF-α*, and *IRGM* were not significantly associated with perianal disease in this population *NOD2 / CARD15* variants (R702W, G908R, 1007 fs) did not show a consistent association with pCD
Yang et al*.* [27] 2014 South Korea	Cohort (NR)	906 CD (390 pCD)	To investigate the association between *TNFSF15* genotypes and the clinical course of CD	*TNFSF15:* rs6478108, rs4574921, rs3810936, rs6478109, and rs7848647	Non-risk allele homozygotes of *TNFSF15* SNP rs6478108 were independent genetic predictors of stenosing and penetrating CD Rs4574921 was identified as an independent genetic predictor for the development of PFCD
Schnitzler et al*.* [29] 2015 Germany	Case-control (NR)	2256 total 1537 IBD (1073 CD; 464 UC) 719 HC	To evaluate the association of rs72796353 with IBD susceptibility and phenotype	*NOD2* SNP: rs72796353 *NOD2* mutations: rs2066844, rs2066845, and rs2066847	The *NOD2* IVS4 + 10 A > C variant showed a significant association with PFCD in the absence of other *NOD2* mutations, suggesting its role as a novel predictor of pCD
Can et al*.* [28] 2015 Turkey	Case-control (NR)	300 total 211 IBD (16 pCD) 89 HC	To investigate the involvement of *TYK2* polymorphisms in patients with IBD	*TYK2:* rs280523, rs2304256, rs280519, and rs280496	The presence of rs2304256 (A) was associated with the presence of pCD (*P* = 0.03) H2/H4 diplotype was observed at higher rates in PFCD
Kaur et al*.* [31] 2016 USA	Case-control (NR)	1721 CD (368 pfCD)	To identify clinical, serological, and genetic factors associated with pCD	Genetic: SNPs in genes related to IBD (*KIF3B, CRTC3, TRAF3IP2, JAZF1, NRIP1, MST1, FUT2, PTGER*); autophagy (*DAPK1*); TNF-α pathways (*NUCB2; DAPK1*); IFN-γ pathways (*DAPK1; NDFIP2*) and extracellular matrix and scaffolding proteins (*USH1C; NDFIP2; TMC07*) and JAK-STAT pathway (*STAT3*) Serological: ASCA, OmpC, and pANCA	Perianal CD was associated with: higher ASCA IgA and OmpC antibody levels known IBD loci, including *KIF3B, CRTC3, TRAF3IP2, JAZF1, NRIP1, MST1, FUT2,* and* PTGER* genes involved in autophagy (*DAPK1*); TNF-α pathways (*NUCB2; DAPK1*); IFN-γ pathways (*DAPK1*; *NDFIP2*); and extracellular matrix and scaffolding proteins (*USH1C; NDFIP2; TMC07*) JAK/Stat pathway (*STAT3*)
Zhang et al*.* [35] 2019 China	Case-control (2012–2016)	750 total 490 CD (250 PFCD) 260 HC	To explore susceptibility genes for CD and pCD in the Han population from South China	Differences in polymorphisms at 90 genetic loci, including SNPs rs72553867 (*IRGM* gene of chr 5), rs4409764 (*NKX2-3* gene of chr 10) and rs3731772 (*AOX1* gene of chr 2), were observed between patients with PFCD, patients with CD, and the HC group	Three SNPs-rs72553867 (*IRGM*), rs4409764 (*NKX2-3*), and rs3731772 (*AOX1*) increase the risk of pCD
Cusato et al*.* [41] 2024 Italy	Case-control (2016–2022)	206 CD (29 PFCD)	To evaluate the association between SNPs in genes encoding enzymes, transporters, and receptors involved in the VD pathway and the occurrence of pCD	Genetic variants in *VDR, CYP27B1, CYP24A1*, and *GC*	The presence of the heterozygous genotype for Apal and BsmI significantly increases the risk of developing pCD
Gisbert-Ferrándiz et al*.* [42] 2024 Spain	Case-control (NR)	115 CD (41 PFCD)	To analyse the association of BsmI, ApaI, TaqI, and FokI SNPs in the *VDR* gene with the clinical characteristics of CD	*VDR* SNP: ApaI (rs7975232)	The aa genotype of the ApaI SNP in the *VDR* gene is associated with a lower risk of PF in CD and with reduced expression of fibrotic and inflammatory markers in intestinal fibroblasts
*Microbiological profile*					
Tozer et al*.* [30] 2015 United Kingdom	Cross-sectional (NR)	51 total 20 PFCD 13 luminal CD 18 CGF	To characterize the microbiota of the fistulous tract and rectal mucosa in patients with CD and CGF	Studied bacteria: *Bifidobacteria* *Clostridium coccoides*-*Eubacterium rectale* cluster *Bacteroides- Prevotella* cluster *Faecalibacterium prausnitzii* *Escherichia coli*	Patients with CD without fistula and those with PFCD had fewer *Bifidobacteria* than those with CGF The tracts of PFCD do not appear to harbor specific bacterial populations Of all the fistula tracts, only one (from a CGF patient) exhibited bacteria that were associated with those of the rectal mucosa
Haac et al*.* [32] 2019 USA	Cross-sectional (NR)	18 PFCD	To characterize the gut microbiota in PFCD using matched stool and fistula samples	*Bacteroides* *Streptococcus* *Bifidobacterium* *Achromobacter* *Corynebacterium*	The microbiota of PF in patients with CD differs from that of stool
Breton et al*.* [38] 2022 USA	Cross-sectional (NR)	108 total 31 PFCD 36 control group CD 41 HC	To define the microbial signature associated with PFCD in children	Bacterial microbiota	The microbial community within CD-related anorectal fistulas is compositionally and functionally unique Resistance genes to ciprofloxacin and metronidazole were detected in all three types of samples
*Immunological profile*					
Ruffolo et al*.* [20] 2008 Italy	Cross-sectional (NR)	41 total 17 pCD (14 PFCD) 7 intestinal CD 17 HC	To assess the cytokine profile in the rectal mucosa of patients with pCD and to examine its relationship with the systemic cytokine profile and inflammatory parameters and the need for surgery	Inflammatory cytokines: TNF-α, IL-1β, IL-6, IL-12, and TGF-β1	IL-1β IL-6, and TNF-α appear to play an important role in pCD Mucosal levels of IL-6 and IL-12 are predictors of recurrence and of need for surgery in patients with pCD
Haddow et al*.* [33] 2019 United Kingdom	Cross-sectional (2014–2015)	61 total 13 PFCD 48 CGF 6 HC	To systematically compare the clinical phenotypes, cytokine profiles, and phosphoprotein profiles of CGF and PFCD	Differences in 30 cytokines and 39 phosphoproteins in PFCD and CGF tissues, as well as in the rectal mucosa	At the internal opening of the fistula, PFCD had higher IL-12p70 levels and lower IL-1RA/IL-1β ratios compared to CGF patients HC rectal mucosa showed higher levels of six phosphoproteins compared to PFCD patients In the fistulous tracts, no differences in cytokines or phosphoproteins levels were observed
Bruckner et al*.* [37] 2021 Switzerland	Translational experimental in vitro (NR)	26 PFCD (15 non-inflamed; 11 inflamed) PBMCs: 9 PFCD; 2 CD; 5 HC	To investigate the role of T-lymphocytes (T cells) in PF pathogenesis	T cells: CD3⁺CD8⁻, CD3⁺CD8⁺, or CD45⁺CD3⁻	PBMCs from patients with inflamed PFCD contained higher numbers of CD3⁺CD8⁻ T lymphocytes and lower numbers of CD3⁺CD8⁺ T cells compared to those from non-inflamed PFCD patients and HC CD3⁺CD8⁻ and CD3⁺CD8⁺ T cells play a crucial role in inflamed PFCD through the secretion of TNF-α and IL-13
Rizzo et al*.* [39] 2023 Italy	Translational experimental in vitro (NR)	29 total 22 PFCD 7 rectal cancer patients	The role of ECM dysregulation in the development of PFCD, compared to normal rectal mucosa from patients with rectal cancer	Profiles of 8172 genes and of *TNFAI6* (gene encoding TSG-6)	By mediating changes in ECM organization, TSG-6 triggers the EMT transcription factor SNAI1 through the activation of mechanosensitive proteins
Levantovsky et al*.* [44] 2024 USA	Cross-sectional (NR)	15 PFCD	To investigate the cellular and molecular mechanisms of PFCD and to identify key biomarkers and potential therapeutic targets	*CHI3L1* OSM (Oncostatin M) Transcription factors JUN/AP-1 Genes associated with cellular senescence Myeloid–stromal crosstalk signal	Enrichment of myeloid cells and fibroblasts in fistula tracts Cellular crosstalk and senescence, confirmed by immunofluorescence CHI3L1 was highly expressed in stromal cells of fistula tissue Differences in *CHI3L1* and OSM expression among monocytes from individuals of African and European ancestry Transcription factor marks associated with stress and inflammatory memory (including JUN/AP-1) and enrichment of genetic loci linked to CD
*Metabonomic profile*					
Adegbola et al*.* [36] 2021 United Kingdom	Cross-sectional (NR)	50 total 20 PFCD 30 CGF	To identify potential biomarkers of disease, that may improve understanding of its pathogenesis	Differences in 41 metabolites between PFCD and CGF, including amino acids, nucleosides, acylcarnitines, and lipids	In PFCD: Increased metabolites: arginine, threonine, tyrosine, lysine, citrulline, proline, methionine, dimethylarginine, homoarginine, O-acetyl-l-carnitine, N-acetyl-lysine, and octanoyl-l-carnitine Decreased metabolites: adenosine, creatinine, hexanoyl-l-carnitine, monoacylglycerol 18:3, S-methyl-5-thioadenosine, decanoyl-l-carnitine, and palmitoyl-l-carnitine Elevated lipids: hexosylceramide (d18:1/16:0) and diglyceride (38:5)

*ASCA* anti-*Saccharomyces cerevisiae* antibodies, *CD* Crohn’s disease, *CGF* cryptoglandular perianal fistula, *Chr* chromosome, *EIMs*, extraintestinal manifestations, *ECM* extracellular matrix, *EMT* epithelial-mesenchymal transition, *HC* healthy controls, *IBD* inflammatory bowel disease, *IFN-γ* interferon-gamma, *IL* interleukin, *JAK/STAT* Janus kinase/signal transducer and activator of transcription, *NR* not reported, *OmpC* outer membrane porin C, *OR* odds ratio, *PBMCs* peripheral blood mononuclear cells, *PF* perianal fistula, *PFCD* perianal fistulising Crohn’s disease, *pCD* perianal Crohn’s disease, *SNP* single-nucleotide polymorphism, *TNF* tumour necrosis factor, *TNF-α* tumour necrosis factor alpha, *UC* ulcerative colitis

**Table 2:** Main characteristics of the diagnostic studies included in the analysis

Study (author, year, country)	Study type (period)	No. of participants	Aims	Biomarker	Outcomes
*Histopathological findings*					
Ramzan et al*.* [16] 2002 USA	Cohort (1990–1994)	82 CD (21 with granuloma; 61 without granuloma) (19 pCD)	To determine whether the presence of granuloma presence is associated with severity of CD, including fistulising behaviour, perianal disease, disease relapses, and EIMs	Presence of granulomas in tissue biopsies examined microscopically in CD	There were no significant differences between the two groups regarding the presence of fistulising or perianal disease, disease severity, recurrence rate, or EIMs
Denoya et al*.* [23] 2011 USA	Cohort (2001–2007)	207 CD (43 PFCD)	To correlate the presence and distribution pattern of granulomas in resected specimens with clinical characteristics and outcomes in patients undergoing surgery for CD	Granuloma	Granulomas were associated with a higher frequency of EIMs and pCD, as well as lower BMI and greater prevalence in younger patients and females
Han et al*.* [43] 2024 China	Case-control (2015–2023)	4430 total (93 PFCD; 4335 with non-CD, non-TB diseases; 2 pulmonary TB)	To assess the histopathological features of granulomas in PFCD	Granuloma	Most PFCD cases show fewer than three granulomas (≤ 3) and no foreign body giant cells Ribbon-like granulomas are only observed in PFCD cases
*Inflammatory profile*					
Stevens et al*.* [34] 2019 The Netherlands	Cross-sectional (2009–2018)	56 total 37 PFCD (19 with ulcers and 18 without) 19 CGF	To determine the diagnostic accuracy of FC to: a) differentiate between PFCD and CGF b) detect mucosal inflammation in pCD	Faecal calprotectin	FC levels were significantly higher in PFCD compared to CGF (µg/g) FC discriminates PFCD from CGF, even in the absence of intestinal ulcers In active pCD, an elevated FC does not accurately predict the presence of ulcers and should be interpreted with caution
Becker et al*.* [40] 2024 The Netherlands	Cross-sectional (2019–2022)	63 total 45 PFCD 18 CGF	To prospectively validate this and to analyse whether increased local fistula calprotectin levels are associated with fistula characteristics	Faecal calprotectin-local calprotectin (from fistula tract scrapings)	PFCD and CGF differ in FC levels Local fistula calprotectin production did not account for this difference, implying FC reflects the luminal condition A correlation exists between calprotectin levels in fistula scrapings and CD fistula severity
Perregaard et al*.* [48] 2025 Denmark	Cross-sectional (2018–2020)	63 total 31 PFCD 32 CGF	To determine the accuracy of FC in distinguishing between PF of CG and CD origin, as well as to compare characteristics in fistula	Faecal calprotectin	FC distinguishes PFCD from CGF, even in clinically managed CD patients with normal endoscopy A FC value ≥ 110 μg/g, combined with clinical suspicion of underlying IBD, should prompt further investigations
Ma et al*.* [47] 2025 China	Case-control (2021–2024)	193 total 96 PFCD 97 CGF	To evaluate NGAL’s potential as a non-invasive diagnostic biomarker for distinguishing between PFCD and CGF and to assess its correlation with disease severity in PFCD	NGAL Faecal calprotectin C-reactive protein Erythrocyte sedimentation rate	NGAL is identified as a promising biomarker for differentiating between PFCD and CGF NGAL levels show a positive correlation with PFCD severity
*Immunological profile*					
Ouboter et al*.* [45] 2024 The Netherlands	Cross-sectional (2019–2023)	Cohort 1: 14 PFCD; 7 non-CD fistula Cohort 2: 10 PFCD; 11 non-CD fistula Cohort 3: 5 PFCD	To characterize the composition and spatial localization of disease-associated immune cells in both types of PF using high-dimensional analyses	HLA- DR⁺CD38⁺ effector Th1/17 cells	Increased activated Th1/17 cells (HLA-DR⁺CD38⁺) distinguish PFCD from CGF and represent a promising blood biomarker CD4⁺ HLA-DR⁺CD38⁺ T cells constitute a potential therapeutic target in CD-associated fistulas
*Metabonomic profile*					
Zhu et al*.* [46] 2024 China	Case-control (July–September 2020)	110 total 55 PFCD 55 simple PF or non-PFCD	To develop a non-invasive detection approach to predict CD in patients with PF	Fifteen and eleven spectral peaks in the urine SERS pattern (phosphoprotein and metabolomics profiles)	The CD group showed increased Raman peaks at 574, 591, 1239, 1276, 1291, and 1499 cm⁻^1^, and decreased peaks at 480, 518, 641, 677, 698, 715, 732, 889, 1181, 1206, and 1566 cm⁻^1^

*BMI* body mass index, *CD* Crohn’s disease, *CGF* cryptoglandular perianal fistula, *EIMs* extraintestinal manifestations, *FC* faecal calprotectin, *IBD* inflammatory bowel disease, *NGAL* neutrophil gelatinase-associated lipocalin, *pCD* perianal Crohn’s disease, *PFCD* perianal fistulising Crohn’s disease, *TB* tuberculosis, *Urine SERS pattern* urine surface-enhanced Raman spectroscopy pattern

Of the 33 included studies, 25 were rated as high quality or low risk of bias, while 6 were rated as moderate quality, based on assessments performed using the NOS. The two translational and experimental studies involving human tissue and in vitro models were rated as high to moderate quality, according to the adapted version of the QUIN scale (Supplementary Table 1).

### Biomarkers of pathogenesis

#### Genetic markers

Several genes have been implicated in the development and clinical course of pCD. Variants in the *NOD2* (*CARD15*) gene were among the first identified as susceptibility factors for CD and have also been investigated in relation to perianal disease phenotypes. Evidence regarding their role in PFCD remains inconsistent. While some studies reported no significant association between *NOD2* mutations and PFCD risk [19, 24]*,* others suggested that specific polymorphisms may influence disease behaviour. For example, variants such as the G allele of rs1363670 (located near the *IL12B* gene) and the T allele of rs12704036 were associated with stricturing disease and early fistula development in males [21]. In addition, the rs72796353 polymorphism, although not associated with overall susceptibility to CD, was associated with PFCD (OR = 5.27; 95% CI 2.75–10.12 vs. NOD2 wild-type carriers; *P* < 0.001), particularly in patients lacking common *NOD2* mutations [29]*.*

Beyond *NOD2,* other loci may contribute to PFCD susceptibility. The rs6908425 variant in *CDKAL1* gene and the absence of *NOD2* mutations were associated with an increased risk of PFCD (OR = 8.86; 95% CI 1.13–69.78; *P* = 0.04 and OR = 0.56; 95% CI 0.38–0.83; *P* = 0.004, respectively), particularly in patients with colonic involvement and smoking habits [21]. Furthermore, *NOD2* mutations were associated with poorer response to antibiotic therapy (metronidazole ± ciprofloxacin; *P* = 0.041), suggesting a potential genetic influence on treatment outcomes [24].

Variants in genes involved in innate immunity, particularly *IRGM* and *NCF4*, were associated with perianal disease. *IRGM* was identified as a genetic risk factor for the fistulising phenotype of CD, including PFCD [22, 35]. In the European population, the rs4958847 polymorphism at *IRGM* was associated with both CD and the development of PF (OR = 1.55; 95% CI 1.01–2.38; *P* = 0.045) [22], with similar findings in the Han Chinese population for rs72553867 [35]. Additional variants included rs4409764 at *NKX2-3* gene and rs3731772 at *AOX1* gene [35].

Multiple genetic loci were linked with more aggressive CD phenotypes. *NCF4* variants were associated with pCD, alongside clinical risk factors such as younger age at diagnosis, complicated intestinal disease, and ileal disease location [26]. Genetic analysis demonstrated a significant association between *NCF4* gene variants when comparing perianal Crohn's disease (pCD) patients with non-perianal CD patients and healthy controls; no significant associations were observed for *IBD5*, TNF-α, or *IRGM* genes [26]***.***

Genetic variability within the JAK-STAT pathway was associated with PFCD (*P* < 0.001) [31], alongside distal colonic involvement, CD-related serology, and family history of IBD. *TYK2* gene variants were also associated with CD and ulcerative colitis (UC) susceptibility, as well as disease phenotype, namely the rs2304256 (A) genotype was more frequent in perineal involvement (*P* = 0.03), whereas the rs2304256 (C) was less frequent. The H2/H4 diplotype (rs280519 AG/rs2304256 AA) was associated with increased risk of perianal involvement [28].

At the *IBD5* locus, *OCTN1 and OCTN2* variants were associated with pCD and penetrating CD but not with overall IBD susceptibility [17]. The *SLC22A5* -207G-C and *SLC22A4* 1672C-T variants were also associated with increased risk of CD and UC (*P* = 0.007). The TC haplotype was associated with PFCD and stricturing-fistulising disease behaviour (*P* = 0.037) and with extensive disease and increased need for immunosuppression in UC [18].

Additional genetic associations included *TNFSF15* rs6478108 (CC), identified as a predictor of CD complications, and rs4574921, which predicted PFCD development (HR = 2.39, 95% CI 1.20–4.73; *P* = 0.013), although treatment effects were not evaluated [27]. *TAGAP* rs212388 (G allele) was associated with reduced risk of perianal septic complications, such as abscesses and fistulas, and lower need for ostomy surgery [25], suggesting a potential protective effect against severe septic forms of PFCD.

*VDR* polymorphisms (*ApaI Aa*, *BsmI Bb*) were associated with development of pCD complications (*P* = 0.01 and *P* = 0.02, respectively) [41], whereas the *Apal aa* genotype was associated with reduced risk of fistulising behaviour [42].

#### Microbiological markers

Microbial dysbiosis was consistently reported in PFCD, particularly in rectal mucosa and fistula-associated samples [30, 32, 38]. PFCD was associated with a reduced bacterial load in the rectal mucosa, with decreased abundances of *Bacteroides* and *Bifidobacterium* compared with CGF [30]. Reduced *Bifidobacterium* abundance in rectal mucosa was also observed in patients with non-fistulising CD compared with CGF [30].

Fistula-associated microbiota differed from stool microbiota, with enrichment of *Achromobacter* and *Corynebacterium* within fistula tracts and a higher abundance of *Bifidobacterium* in stool samples [32]. Metagenomic studies in paediatric CD reported increased *Proteobacteria*, depletion of butyrate-producing bacteria, and the presence of antibiotic resistance genes in fistula-associated microbiota [38].

Overall, PFCD was associated with a reduction in commensal bacteria, enrichment of *Proteobacteria* and other opportunistic taxa, and altered microbial functional profiles [30, 32, 38].

#### Immunological markers

Elevated levels of IL-1β, IL-6, and TNF-α were reported in the rectal mucosa of patients with pCD and were associated with higher disease activity and surgical need. PFCD showed greater severity and anatomical complexity than idiopathic PF [33]. Tissue biopsies, cytokines, and phosphoprotein profiling revealed elevated IL-12p70 levels and a reduced IL-1RA/IL-1β ratio, indicating impaired anti-inflammatory regulation in PFCD [33]*.*

T-cell subsets (CD3 + CD8- and CD3 + CD8 +) were implicated in pathogenesis, associated with TNF-α overexpression and epithelial-mesenchymal transition (EMT), and involved IL-13 and IL-22 [37].

Extracellular matrix dysregulation was identified in PFCD, with increased TNFAIP6, altered ECM organisation, and upregulation of integrins, metalloproteinases, and collagen deposition [39].

Immune-stromal interactions were observed in PFCD, involving myeloid cells and fibroblasts, alongside senescence pathways and epigenetic changes (JUN/AP-1). Differential expression of CHI3L1 and OSM was reported between monocytes from individuals of African and European ancestry [44].

#### Metabolic markers

Distinct metabolite profiles were observed between PFCD and CGF [36]. Nineteen metabolites differed significantly between the two conditions. PFCD was associated with increased levels of arginine, threonine, tyrosine, lysine, citrulline, proline, methionine, dimethylarginine, homoarginine, O-acetyl-l-carnitine, N-acetyl-lysine, and octanoyl-l-carnitine. Lower levels of adenosine, creatinine, hexanoyl-l-carnitine, monoacylglycerol 18:3, S-methyl-5-thioadenosine, decanoyl-l-carnitine, and palmitoyl-l-carnitine were observed in PFCD than in CGF. Higher levels of hexosylceramide and diglyceride were also identified in PFCD [36].

### Diagnostic biomarkers

#### Histopathological markers

The prognostic value of granulomas in CD remains uncertain. Ramzan et al. [16] reported no association between granuloma presence and disease severity, pCD, extraintestinal manifestations (EIMs), or recurrence, indicating limited prognostic utility and supporting their role primarily in differential diagnosis. In contrast, Denoya et al. [23] identified distinct clinical associations in patients with granulomas in surgical specimens, including higher prevalence in females (*P* = 0.017), younger age at presentation (*P* < 0.001), lower body mass index (*P* = 0.037), increased incidence of pCD (*P* = 0.012), and greater occurrence of EIMs (*P* = 0.026). More recently, Han et al. [43] described a distinct granuloma subtype in PFCD, characterised by a band-like architecture, smaller size, and absence of multinucleated giant cells, which may facilitate differentiation between CD-related and non-CD-related disease.

#### Inflammatory markers

Faecal calprotectin (FC) is a useful non-invasive biomarker for differentiating PFCD from non-Crohn’s fistulas. Its effectiveness has been demonstrated using a cut-off value of ≥ 150 μg/g, achieving a sensitivity of 81% and a specificity of 89% [34]. FC levels were higher in PFCD compared with CGF (354.3 vs. 47.3 μg/g, *P* = 0.003), and calprotectin measured directly in the fistulous tract was correlated with clinical and radiological severity, suggesting potential prognostic relevance [40]. In addition, FC was validated as a screening tool in patients with PF (OR = 12.5; 95% CI 3.77–41.4; *P* < 0.0001), with a cut-off of ≥ 110 μg/g associated with CD (sensitivity = 76% and specificity = 80%), even in patients without endoscopic activity or receiving treatment [48]*.*

Serum neutrophil gelatinase-associated lipocalin (NGAL) demonstrated high diagnostic performance (AUC = 0.927; 95% CI, 0.890–0.964), exceeding that reported for FC. NGAL also correlated with clinical parameters and inflammatory markers, supporting its utility for both diagnosis and assessment of PFCD severity [47].

#### Immunological and metabolomic markers

A specific population of activated CD4⁺ T cells (HLA-DR⁺CD38⁺) with a Th1/Th17 profile was identified and showed a significant association with PFCD. These cells were detected in both fistula tissue and peripheral blood. Their interaction with CCR6⁺ B lymphocytes and macrophages suggested a distinct immunological mechanism, with potential diagnostic and therapeutic implications [45].

Urinary Raman spectroscopy combined with machine learning demonstrated discriminatory ability between PFCD and CGF, with sensitivity = 71%, specificity = 80%, and accuracy 76% (the leave-one-patient-out cross-validation), indicating systemic metabolic alterations. Significant differences in 11 spectral peaks were observed, supporting the potential of a urinary biochemical signature as a complementary diagnostic tool [46].

## Discussion

In this systematic review of 33 articles, we provide a comprehensive synthesis of the current literature on biomarkers involved in the pathogenesis and diagnosis of PFCD. Overall, the studies reviewed consistently demonstrate that PFCD is a biologically heterogeneous and multidimensional condition, arising from the dynamic interaction among genetic predisposition, environmental exposures, dysregulated immune responses, metabolic alterations, and processes of tissue remodelling. This complexity underpins the marked variability in disease course, severity, and therapeutic response among patients.

Genetic studies further reinforce the concept that CD results from a complex interplay between hereditary susceptibility and environmental factors, rather than being driven by a single gene. Despite the growing number of polymorphisms associated with PFCD, most exhibit small effect sizes and limited predictive value when considered individually, which restricts their clinical utility as independent biomarkers. Nevertheless, several genetic variants have been consistently associated with specific clinical phenotypes, particularly perianal disease, supporting a potential role for genetic profiling in risk stratification.

In European cohorts, polymorphisms in the *OCTN1/2* (*SLC22A4/5*) [17, 18], *IRGM* (rs4958847) [22], *CDKAL1* (rs6908425) [21], and *NOD2* (rs72796353) genes [29] have been reproducibly linked to perianal involvement. Similarly, in Asian populations, significant associations have been identified with the *TNFSF15* rs4574921 (CC genotype) gene in Korean patients [27], as well as variants in *IRGM* (rs72553867), *NKX2-3* (rs4409764), and *AOX1* (rs3731772) in Chinese patients [35]. Additionally, variants in the *NCF4* gene have been reported in cohorts from New Zealand [26], further supporting the contribution of pathways related to innate immune responses in the development of perianal disease.

Beyond associations with individual genes, broader signalling pathways appear to play a key role in disease aggressiveness. The JAK-STAT pathway, particularly mediated by variants in the *TYK2* (rs2304256) gene [28], has been associated with more severe and fistulising forms of perianal disease [31], highlighting its relevance in the regulation of inflammation and immune responses. In contrast, variants in the *TAGAP* gene appear to confer a protective effect against severe septic perianal complications, suggesting a modulatory role in clinical expression and disease progression [25].

Consistently, the most robust associations involve variants in genes related to innate immunity and immune regulation, namely *NOD2* (CARD15), *IRGM*, *NCF4*, and components of the JAK-STAT pathway, particularly *TYK2*, reinforcing the central role of immune dysfunction in the development of more severe perianal phenotypes. Associations with intermediate levels of evidence have also been reported, often population-dependent, including variants in *OCTN1/2* (IBD5) and *TNFSF15*, which are associated with more aggressive phenotypes and fistulising complications, particularly in specific ethnic contexts.

Some emerging genetic markers, such as *NKX2-3* and *AOX1*, remain insufficiently validated and require confirmation in independent cohorts before any potential clinical application [35]. Similarly, the role of the vitamin D receptor (*VDR*) pathway remains controversial, with inconsistent findings that may reflect methodological differences as well as the influence of environmental and nutritional factors [41, 42].

Substantial inter-study heterogeneity—encompassing ethnic variation, phenotypic definitions, and methodological differences—limits direct comparability and likely underlies the inconsistencies across findings. Nevertheless, genotyping of specific SNPs appears promising for predicting more severe phenotypes, reinforcing the emerging role of precision medicine in risk stratification and the personalised management of CD.

The gut microbiota also plays a key role in the pathogenesis of PFCD. According to the cryptoglandular theory, chronic bacterial infection is considered the main factor underlying the development and persistence of fistulas [5]. However, some authors have questioned this hypothesis, arguing that bacterial presence in both CGF and PFCD is often limited and may not be decisive for disease maintenance, with the composition and function of the microbiota potentially being more relevant [30, 49].

In this context, recent studies suggest the presence of phenotype-specific microbial signatures, with reduced microbial diversity and shifts in key taxa associated with disease severity [50].

 Findings such as the enrichment of *Achromobacter* and *Corynebacterium* in fistula tracts and increased faecal *Bifidobacterium* levels [32], as well as the higher abundance of *Proteobacteria* and antibiotic resistance genes in the fistula microbiome [38], support a role for niche-specific dysbiosis. However, the predominance of observational study designs and the lack of functional analyses limit causal interpretation.

From a mechanistic perspective, microbial components such as muramyl dipeptide (MDP) may contribute to fistula formation in CD by inducing EMT. This process leads to the activation of inflammatory mediators, namely TNF-α, TGF-β, SNAIL1, and IL-13 [51, 52], which in turn promote the upregulation of matrix metalloproteinases MMP-3 and MMP-9, favouring extracellular matrix degradation and the perpetuation of fistulous lesions [53].

Beyond the bacterial component, emerging evidence also suggests a role for the mycobiota. The recent identification of the fungus *Debaryomyces hansenii* in inflamed intestinal tissue represents an important advance and suggests that fungal dysbiosis may act not only as an inflammatory trigger but also as a factor contributing to chronicity [54].

Overall, studies indicate relatively consistent trends of intestinal dysbiosis in PFCD; however, variability in sampling methods, study populations, and sequencing approaches continues to hinder the reliable identification of microbial signatures.

In the immunological context, the pathogenesis of PFCD involves complex inflammatory and structural mechanisms that extend beyond the classical TNF-α pathway [37, 51, 55]. Previous studies have shown elevated levels of multiple pro-inflammatory cytokines, including IL-1β, IL-6, TNF-α, and IL-12p70 [20, 33, 56, 57], which are associated with increased disease activity and fistulising complexity. However, inconsistent correlations with clinical outcomes limit their current clinical applicability.

In CGF, several studies have demonstrated elevated pro-inflammatory cytokine profiles in fistula tissue. However, these findings should be interpreted with caution because of methodological heterogeneity, differences in study populations, and variability in sampling sites. Increased levels of IL-1β and IL-8 have been reported in chronic fistula tissue compared with healthy controls, with IL-1β predominantly localised in the distal regions of the tract and IL-8 more abundant proximally [56]. Similarly, elevated expression of IL-1β, IL-8, IL-12p40, and TNF-α has been identified in the distal fistula tract, although these increases did not consistently correlate with specific clinical outcomes [52]. Additionally, increased IL-1β and IL-8 expression has been observed in chronic CGF tissues, along with histological features suggestive of EMT, a process implicated in tissue remodelling and fistula persistence [58]. Although these findings highlight a complex inflammatory microenvironment, variability across studies limits their direct clinical translation.

Despite these advances, significant knowledge gaps remain, including the precise role of IL-22 and population-specific differences in cytokine expression and molecular pathways. Overall, the evidence supports a multifactorial inflammatory environment but remains insufficient to justify broad therapeutic expansion beyond the TNF-α pathway, emphasising the need for more robust mechanistic and clinical studies.

Metabolomics has emerged as an innovative tool for characterising PFCD. Alterations such as increased dimethylarginine and diacylglycerols [36, 59] alongside reduced decanoyl-l-carnitine, suggest that metabolic disturbances may promote both EMT and tissue remodelling. However, current evidence does not establish causality, and these alterations may, at least in part, reflect chronic inflammation. The observation of elevated diacylglycerol levels in fistula tissue supports previous systemic metabolic findings [60]; however, it does not clarify the directionality of these alterations and instead reinforces the hypothesis that altered metabolic pathways are involved in fistula pathophysiology.

In parallel, surface-enhanced Raman spectroscopy (SERS) has recently identified highly sensitive and specific urinary biomarkers [46], making urine a valuable non-invasive option for early diagnosis and monitoring of PFCD activity—particularly important for this frequently underdiagnosed phenotype. However, these findings remain at an exploratory stage and require validation in larger, independent cohorts.

More recently, studies in cell and molecular biology have shown that PFCD results from a complex crosstalk between immune and stromal cells, marked by senescence, epigenetic alterations, and population-specific differences [44]. These mechanisms indicate that fistula formation results not only from chronic inflammation but also from profound changes in tissue architecture and cellular behaviour, reflecting a broader process of tissue reprogramming. Although these findings expand current understanding, their predominantly descriptive nature limits causal inference in vivo, and the hierarchy of mechanisms underlying fistula formation remains unclear.

Although granulomas are relevant for histological diagnosis, available evidence indicates that their prognostic value is limited [16, 23, 43]. More recent studies suggest that specific granuloma features may help identify clinically more complex cases [43]; however, these findings remain isolated and lack external validation. Therefore, their prognostic value and therapeutic utility remain uncertain, and they should be interpreted with caution, at most as an adjunct within the overall clinical context.

Among inflammatory markers, faecal calprotectin (FC) remains a valuable tool for distinguishing PFCD from CGF and for assessing local inflammatory activity [34, 40, 48]. Serum NGAL has also emerged as a promising non-invasive marker of perianal involvement in CD patients [47]; however, the evidence remains preliminary.

Recent findings in immunology and metabolomics, including the identification of specific T-cell subpopulations and alterations in urinary profiles, suggest that PFCD involves distinct biological mechanisms with potential diagnostic and therapeutic value [46]. Nevertheless, these findings require validation in larger cohorts using standardised methodologies before clinical implementation.

Across domains, evidence base remains largely observational and heterogeneous, limiting causal inference and highlighting the need for prospective, standardised validation studies. Collectively, advances in genetics, microbiota, immunology, metabolomics, and cellular biology underscore the multidimensional nature of PFCD (Table 3). Biomarkers derived from these domains may improve diagnostic accuracy, refine risk stratification, and support personalised therapeutic decision-making.

**Table 3:** Factors associated with risk and activity of PFCD

Domain	Key factors/impact	References
Genetics	Variants [OCTN1/2 (*SLC22A4/5*), *NOD2, CDKAL1, TNFSF15, TYK2, IGRM, AOX1, NKX2-3, VDR*] → increased risk of perianal phenotype; population differences (e.g., CHI3L1, OSM) modulate susceptibility	[17, 18, 21, 22, 26–29, 31, 35, 41]
Microbiota	*Achromobacter, Corynebacterium,* Proteobacteria, *Debaryomyces* → inflammation and fistula maintenance	[32, 38, 53]
Inflammation	IL-1β, IL-6, TNF-α, IL-12p70, IL-13, IL-22, EMT → high activity and complex fistulas; crosstalk between immune and stromal cells	[20, 33, 37, 39, 50, 54–56]
Cellular senescence/ epigenetics	Senescence pathways, epigenetic modifications, transcription factor footprints (JUN/AP-1) → memory of inflammation and persistent fibrotic/fistulising response	[44]
Tissue remodelling/ metabolomics	MMP-3/9, dimethylarginine, diacylglycerols → fistula persistence and progression; fibroblast activation and ECM remodelling	[36, 50–52, 58, 59]
Diagnostic biomarkers	Faecal calprotectin, serum NGAL, urinary SERS → non-invasive assessment of activity	[34, 40, 46–48]
Histology	Recent morphological features and presence of granulomas → differentiation of complex fistulas	[16, 23, 43]

This review employed a structured methodology to identify, select, and critically appraise relevant studies, thereby minimising investigator bias and ensuring an objective synthesis of the available evidence. However, several limitations should be acknowledged. There is considerable heterogeneity among the biomarkers evaluated across the included studies, which hinders comparability and reduces the consistency of conclusions regarding potential microbial or inflammatory signatures. In addition, the included studies were conducted in different populations, which may have introduced selection bias and limited the generalisability of the findings to broader clinical settings. Most studies were retrospective and did not adequately control for confounding factors, which may have influenced the observed associations between biomarkers and disease phenotypes. Consequently, the inability to perform a meta-analysis further limits the robustness and precision of the conclusions, thereby reducing their clinical applicability, particularly regarding the use of biomarkers for diagnosis, stratification, and prognosis.

## Future directions

Progress toward clinically actionable biomarkers for PFCD will depend on well-designed, large-scale, multicentre prospective studies, ideally including robust validation cohorts to ensure reproducibility and generalisability. Standardisation of biomarker assessment methodologies will be essential to facilitate comparability across studies. In parallel, the integration of multi-omic approaches—including genomics, transcriptomics, proteomics, and metabolomics—may provide a more comprehensive understanding of the molecular mechanisms underlying PFCD. Longitudinal study designs will be particularly important to clarify temporal dynamics and disease evolution. Further work should also focus on the development of integrated biomarker panels combining genetic, microbial, immunological, and metabolic data, which may improve patient stratification and support more precise clinical decision-making. Ultimately, these approaches are expected to refine disease phenotyping and support the transition towards personalised, multimodal therapeutic strategies in PFCD.

## Conclusions

PFCD is a biologically complex and heterogeneous condition driven by the interplay of genetic susceptibility, immune dysregulation, microbial imbalance, metabolic alterations, and tissue remodelling. This multifactorial pathophysiology underlies its variable clinical behaviour and therapeutic response. Although a wide range of biomarkers has been associated with disease phenotype and activity, the current evidence remains largely observational and heterogeneous, limiting their immediate clinical applicability.

Overall, emerging data support the potential value of integrated biomarker approaches for improving disease characterisation and stratification in PFCD, although their incorporation into routine clinical practice requires further validation.

## Supplementary Information

Below is the link to the electronic supplementary material.

## Data Availability

This systematic review did not generate new datasets. All data were extracted from published studies identified through systematic searches of PubMed and Scopus and are available within the original articles cited in the reference list.

## Abbreviations

AOX1: Aldehyde oxidase 1
AUC: Area under the curve
CARD15: Caspase recruitment domain-containing protein 15
CDKAL1: Cyclin-dependent kinase 5 regulatory subunit associated protein 1-like 1
CGF: Cryptoglandular fistulas
CHI3L1: Chitinase-3-like protein 1
ECM: Extracellular matrix
EIMs: Extraintestinal manifestations
EMT: Epithelial-mesenchymal transition
FC: Faecal calprotectin
IRGM: Immunity-related GTPase family M
JAK-STAT: Janus kinases-signal transducers and activators of transcription
JUN/AP-1: Jun proto-oncogene/activator protein 1
MMPs: Matrix metalloproteinases
NGAL: Neutrophil gelatinase-associated lipocalin
NKX2-3: NK2 homeobox 3
NOD2: Nucleotide-binding oligomerization domain-containing protein 2
NOS: Newcastle–Ottawa scale
OCTN1/2: Organic cation transporter
OSM: Oncostatin M
pCD: Perianal Crohn’s disease
PF: Perianal fistula
PFCD: Perianal fistulas in Crohn’s disease
QUIN: Quality assessment tool for in vitro studies
SERS: Surface-enhanced Raman spectroscopy
SLC22A4/5: Solute carrier family 22 member 4/5
SNAIL1: Snail family transcriptional repressor 1
SNP: Single nucleotide polymorphism
TAGAP: T-cell activation Rho GTPase activating protein
Th1/17: T helper 1/17 cells
TNFAIP6: Tumour necrosis factor alpha-induced protein 6
TNFSF15: Tumour necrosis factor superfamily member 15

## Declarations

### Publication statement

We confirm that this manuscript including related data, figures, and tables has not been previously published and is not under consideration elsewhere.

## References

[1] Feuerstein JD, Ho EY, Shmidt E et al (2021) AGA clinical practice guidelines on the medical management of moderate to severe luminal and perianal fistulizing Crohn’s disease. Gastroenterology 160(7):2496–2508. 10.1053/j.gastro.2021.04.022 34051983 PMC8988893

[2] Mei Z, Wang Q, Zhang Y et al (2019) Risk factors for recurrence after anal fistula surgery: a meta-analysis. Int J Surg 69:153–164. 10.1016/j.ijsu.2019.08.003 31400504

[3] Bondurri A (2022) Epidemiology of anal fistula and abscess. In: Ratto C, Parello A, Litta F, De Simone V, Campennì P (eds) Anal fistula and abscess. Springer International Publishing, Cham, pp 3–11

[4] Zhou Z, Ouboter LF, Peeters K, Peeters KCMJ et al (2023) Crohn’s disease-associated and cryptoglandular fistulas: differences and similarities. J Clin Med. 10.3390/jcm12020466 36675403 PMC9860571

[5] Parks AG (1961) Pathogenesis and treatment of fistuila-in-ano. Br Med J 1(5224):463–469. 10.1136/bmj.1.5224.463 13732880 PMC1953161

[6] Sugrue J, Nordenstam J, Abcarian H et al (2017) Pathogenesis and persistence of cryptoglandular anal fistula: a systematic review. Tech Coloproctol 21(6):425–432. 10.1007/s10151-017-1645-5 28620877

[7] Zhu J, Wang Q, Mei Z (2021) Preliminary study on the pathogenesis of anal fistula. medRxiv:2021.2004.2015.21254769. 10.1101/2021.04.15.21254769.

[8] Bataille F, Rohrmeier C, Bates R et al (2008) Evidence for a role of epithelial mesenchymal transition during pathogenesis of fistulae in Crohn’s disease. Inflamm Bowel Dis 14(11):1514–1527. 10.1002/ibd.20590 18626977

[9] Kirkegaard T, Hansen A, Bruun E, Brynskov J (2004) Expression and localisation of matrix metalloproteinases and their natural inhibitors in fistulae of patients with Crohn’s disease. Gut 53(5):701–709. 10.1136/gut.2003.017442 15082589 PMC1774029

[10] Tozer PJ, Burling D, Gupta A, Phillips RK, Hart AL (2011) Review article: medical, surgical and radiological management of perianal Crohn’s fistulas. Aliment Pharmacol Ther 33(1):5–22. 10.1111/j.1365-2036.2010.04486.x 21083581

[11] Adamina M, Minozzi S, Warusavitarne J et al (2024) ECCO guidelines on therapeutics in Crohn’s disease: surgical treatment. J Crohns Colitis 18(10):1556–1582. 10.1093/ecco-jcc/jjae089 38878002

[12] McCurdy JD, Parlow S, Dawkins Y et al (2020) Tumor necrosis factor inhibitors may have limited efficacy for complex perianal fistulas without luminal Crohn’s disease. Dig Dis Sci 65(6):1784–1789. 10.1007/s10620-019-05905-y 31642006

[13] Gold SL, Cohen-Mekelburg S, Schneider Y, Steinlauf A (2018) Perianal fistulas in patients with Crohn’s disease, part 2: surgical, endoscopic, and future therapies. Gastroenterol Hepatol (N Y) 14(9):521–528 30364296 PMC6194657

[14] Page MJ, McKenzie JE, Bossuyt PM et al (2021) The PRISMA 2020 statement: an updated guideline for reporting systematic reviews. BMJ 372:n71. 10.1136/bmj.n71 33782057 PMC8005924

[15] Stang A (2010) Critical evaluation of the Newcastle-Ottawa scale for the assessment of the quality of nonrandomized studies in meta-analyses. Eur J Epidemiol 25(9):603–605. 10.1007/s10654-010-9491-z 20652370

[16] Ramzan NN, Leighton JA, Heigh RI, Shapiro MS (2002) Clinical significance of granuloma in Crohn’s disease. Inflamm Bowel Dis 8(3):168–173. 10.1097/00054725-200205000-00002 11979136

[17] Vermeire S, Pierik M, Hlavaty T et al (2005) Association of organic cation transporter risk haplotype with perianal penetrating Crohn’s disease but not with susceptibility to IBD. Gastroenterology 129(6):1845–1853. 10.1053/j.gastro.2005.10.006 16344053

[18] Palmieri O, Latiano A, Valvano R et al (2006) Variants of OCTN1-2 cation transporter genes are associated with both Crohn’s disease and ulcerative colitis. Aliment Pharmacol Ther 23(4):497–506. 10.1111/j.1365-2036.2006.02780.x 16441470

[19] Jakubowska L, Kaczmarek M, Hoppe-Gołȩbiewska J et al (2008) CARD15/NOD2 G908R, DLG5 R30Q and OCTN1 F503L polymorphisms according to localization of intestinal and extra-intestinal symptoms of the disease among patients with Crohn’s disease in Polish population. Gastroenterologia Polska 15(5):293–296

[20] Ruffolo C, Scarpa M, Faggian D et al (2008) Cytokine network in rectal mucosa in perianal Crohn’s disease: relations with inflammatory parameters and need for surgery. Inflamm Bowel Dis 14(10):1406–1412. 10.1002/ibd.20486 18452203

[21] Henckaerts L, Van Steen K, Verstreken I et al (2009) Genetic risk profiling and prediction of disease course in Crohn’s disease patients. Clin Gastroenterol Hepatol 7(9):972-980.e972. 10.1016/j.cgh.2009.05.001 19422935

[22] Latiano A, Palmieri O, Cucchiara S et al (2009) Polymorphism of the IRGM gene might predispose to fistulizing behavior in Crohn’s disease. Am J Gastroenterol 104(1):110–116. 10.1038/ajg.2008.3 19098858

[23] Denoya P, Canedo J, Berho M et al (2011) Granulomas in Crohn’s disease: does progression through the bowel layers affect presentation or predict recurrence? Colorectal Dis 13(10):1142–1147. 10.1111/j.1463-1318.2010.02421.x 20860713

[24] Freire P, Portela F, Donato MM, Ferreira M, Andrade P, Sofia C (2011) CARD15 mutations and perianal fistulating Crohn’s disease: correlation and predictive value of antibiotic response. Dig Dis Sci 56(3):853–859. 10.1007/s10620-010-1331-1 20632099

[25] Connelly TM, Sehgal R, Berg AS et al (2012) Mutation in TAGAP is protective of anal sepsis in ileocolic Crohn’s disease. Dis Colon Rectum 55(11):1145–1152. 10.1097/DCR.0b013e3182676931 23044675

[26] Eglinton TW, Roberts R, Pearson J et al (2012) Clinical and genetic risk factors for perianal Crohn’s disease in a population-based cohort. Am J Gastroenterol 107(4):589–596. 10.1038/ajg.2011.437 22158027

[27] Yang DH, Yang SK, Song K et al (2014) TNFSF15 is an independent predictor for the development of Crohn’s disease-related complications in Koreans. J Crohns Colitis 8(10):1315–1326. 10.1016/j.crohns.2014.04.002 24835165

[28] Can G, Tezel A, Gürkan H et al (2015) Tyrosine kinase-2 gene polymorphisms are associated with ulcerative colitis and Crohn’s disease in Turkish Population. Clin Res Hepatol Gastroenterol 39(4):489–498. 10.1016/j.clinre.2015.01.005 25744728

[29] Schnitzler F, Friedrich M, Wolf C et al (2015) The NOD2 single nucleotide polymorphism rs72796353 (IVS4+10 A>C) is a predictor for perianal fistulas in patients with Crohn’s disease in the absence of other NOD2 mutations. PLoS ONE 10(7):e0116044. 10.1371/journal.pone.0116044 26147989 PMC4493062

[30] Tozer PJ, Rayment N, Hart AL et al (2015) What role do bacteria play in persisting fistula formation in idiopathic and Crohn’s anal fistula? Colorectal Dis 17(3):235–241. 10.1111/codi.12810 25359567

[31] Kaur M, Panikkath D, Yan X et al (2016) Perianal Crohn’s disease is associated with distal colonic disease, stricturing disease behavior, IBD-associated serologies and genetic variation in the JAK-STAT pathway. Inflamm Bowel Dis 22(4):862–869. 10.1097/MIB.0000000000000705 26937622 PMC5220246

[32] Haac BE, Palmateer NC, Seaton ME, Van YR, Fraser CM, Bafford AC (2019) A distinct gut microbiota exists within Crohn’s disease-related perianal fistulae. J Surg Res 242:118–128. 10.1016/j.jss.2019.04.032 31075656

[33] Haddow JB, Musbahi O, MacDonald TT, Knowles CH (2019) Comparison of cytokine and phosphoprotein profiles in idiopathic and Crohn’s disease-related perianal fistula. World J Gastrointest Pathophysiol 10(4):42–53. 10.4291/wjgp.v10.i4.42 31750007 PMC6854389

[34] Stevens TW, D’Haens GR, Duijvestein M, Bemelman WA, Buskens CJ, Gecse KB (2019) Diagnostic accuracy of faecal calprotectin in patients with active perianal fistulas. United Eur Gastroenterol J 7(4):496–506. 10.1177/2050640619834464 PMC6488796 31065367

[35] Zhang M, Wang X, Jiang X et al (2019) Polymorphisms of the TNF gene and three susceptibility loci are associated with Crohn’s disease and perianal fistula Crohn’s disease: a study among the Han population from South China. Med Sci Monit 25:9637–9650. 10.12659/msm.917244 31844038 PMC6929548

[36] Adegbola SO, Sarafian M, Sahnan K et al (2021) Differences in amino acid and lipid metabolism distinguish Crohn’s from idiopathic/cryptoglandular perianal fistulas by tissue metabonomic profiling and may offer clues to underlying pathogenesis. Eur J Gastroenterol Hepatol 33(12):1469–1479. 10.1097/meg.0000000000001976 33337668

[37] Bruckner RS, Spalinger MR, Barnhoorn MC et al (2021) Contribution of CD3+CD8- and CD3+CD8+ T Cells to TNF-α overexpression in Crohn disease-associated perianal fistulas and induction of epithelial-mesenchymal transition in HT-29 Cells. Inflamm Bowel Dis 27(4):538–549. 10.1093/ibd/izaa240 33146394

[38] Breton J, Tanes C, Tu V et al (2022) A microbial signature for paediatric perianal Crohn’s disease. J Crohns Colitis 16(8):1281–1292. 10.1093/ecco-jcc/jjac032 35211723

[39] Rizzo G, Rubbino F, Elangovan S et al (2023) Dysfunctional extracellular matrix remodeling supports perianal fistulizing Crohn’s disease by a mechanoregulated activation of the epithelial-to-mesenchymal transition. Cell Mol Gastroenterol Hepatol 15(3):741–764. 10.1016/j.jcmgh.2022.12.006 36521659 PMC9898761

[40] Becker MAJ, Stevens TW, de Voogd FAE et al (2024) Clinical relevance of calprotectin in patients with perianal fistulas in Crohn’s disease and cryptoglandular fistulas. United Eur Gastroenterol J. 10.1002/ueg2.12622 PMC11999044 39680482

[41] Cusato J, Cafasso C, Antonucci M et al (2024) Correlation between polymorphisms of vitamin D metabolism genes and perianal disease in Crohn’s disease. Biomedicines. 10.3390/biomedicines12020320 38397922 PMC10886824

[42] Gisbert-Ferrándiz L, Llau J, Ortiz-Masia D et al (2024) ApaI polymorphism in the vitamin D receptor gene decreases the risk of perianal fistulas in Crohn’s disease. Nutrients. 10.3390/nu16203485 39458479 PMC11510363

[43] Han YN, Gu QR, Wu YC, Topatana W, Jiang ZN (2024) Granulomas without foreign body giant cells in perianal fistula tissue suggest Crohn’s disease. J Dig Dis 25(8):484–489. 10.1111/1751-2980.13312 39350692

[44] Levantovsky RM, Tastad C, Zhang J et al (2024) Multimodal single-cell analyses reveal mechanisms of perianal fistula in diverse patients with Crohn’s disease. Med 5(8):886-908.e811. 10.1016/j.medj.2024.03.021 38663404 PMC11317226

[45] Ouboter LF, Lindelauf C, Jiang Q et al (2024) Activated HLA-DR+CD38+ effector Th1/17 cells distinguish Crohn’s disease-associated perianal fistulas from cryptoglandular fistulas. Inflamm Bowel Dis 30(11):2146–2161. 10.1093/ibd/izae103 38776553 PMC11812577

[46] Zhu Y, Xu W, Liu Z et al (2024) Surface-enhanced Raman spectroscopy analysis reveals biochemical difference in urine of patients with perianal fistula. Asian J Surg 47(1):140–146. 10.1016/j.asjsur.2023.05.137 37308382

[47] Ma K, Li Y, Wu J et al (2025) Differential Diagnosis value of neutrophil gelatinase associated lipocalin as a noninvasive biomarker in perianal fistulizing Crohn’s disease. J Inflamm Res 18:4075–4086. 10.2147/jir.S504213 40125092 PMC11930251

[48] Perregaard H, Pust F, Nordholm-Carstensen A (2025) Faecal calprotectin as a non-invasive marker of Crohn’s disease in anal fistulas. Colorectal Dis 27(2):e70026. 10.1111/codi.70026 39952904

[49] van Onkelen RS, Mitalas LE, Gosselink MP, van Belkum A, Laman JD, Schouten WR (2013) Assessment of microbiota and peptidoglycan in perianal fistulas. Diagn Microbiol Infect Dis 75(1):50–54. 10.1016/j.diagmicrobio.2012.09.012 23102557

[50] Goren I, Ian W, Reshef L et al (2021) P683 Patients with newly diagnosed fistulizing perianal Crohn’s disease have a distinct microbial signature. J Crohns Colitis 15(Supplement_1):S602–S602. 10.1093/ecco-jcc/jjab076.803

[51] Frei SM, Pesch T, Lang S et al (2013) A role for tumor necrosis factor and bacterial antigens in the pathogenesis of Crohnʼs disease–associated fistulae. Inflamm Bowel Dis 19(13):2878–2887. 10.1097/01.MIB.0000435760.82705.23 24189042

[52] van Onkelen RS, Gosselink MP, van Meurs M, Melief MJ, Schouten WR, Laman JD (2016) Pro-inflammatory cytokines in cryptoglandular anal fistulas. Tech Coloproctol 20(9):619–625. 10.1007/s10151-016-1494-7 27402195 PMC5003909

[53] Scharl M, Weber A, Furst A et al (2011) Potential role for SNAIL family transcription factors in the etiology of Crohnʼs disease-associated fistulae. Inflamm Bowel Dis 17(9):1907–1916. 10.1002/ibd.21555 21830269

[54] Jain U, Ver Heul AM, Xiong S et al (2021) Debaryomyces is enriched in Crohn’s disease intestinal tissue and impairs healing in mice. Science 371(6534):1154–1159. 10.1126/science.abd0919 33707263 PMC10114606

[55] Scharl M, Rogler G (2014) Pathophysiology of fistula formation in Crohn’s disease. World J Gastrointest Pathophysiol 5(3):205–212. 10.4291/wjgp.v5.i3.205 25133023 PMC4133520

[56] Kiehne K, Fincke A, Brunke G, Lange T, Folsch UR, Herzig KH (2007) Antimicrobial peptides in chronic anal fistula epithelium. Scand J Gastroenterol 42(9):1063–1069. 10.1080/00365520701320489 17710671

[57] Scharl M, Frei S, Pesch T et al (2013) Interleukin-13 and transforming growth factor beta synergise in the pathogenesis of human intestinal fistulae. Gut 62(1):63–72. 10.1136/gutjnl-2011-300498 22287592

[58] Ratto C, Litta F, Lucchetti D et al (2016) Immunopathological characterization of cryptoglandular anal fistula: a pilot study investigating its pathogenesis. Colorectal Dis 18(12):O436–O444. 10.1111/codi.13527 27649390

[59] Owczarek D, Cibor D, Mach T (2010) Asymmetric dimethylarginine (ADMA), symmetric dimethylarginine (SDMA), arginine, and 8-iso-prostaglandin F2α (8-iso-PGF2α) level in patients with inflammatory bowel diseases. Inflamm Bowel Dis 16(1):52–57. 10.1002/ibd.20994 19575355

[60] Santoru ML, Piras C, Murgia A et al (2017) Cross sectional evaluation of the gut-microbiome metabolome axis in an Italian cohort of IBD patients. Sci Rep 7(1):9523. 10.1038/s41598-017-10034-5 28842640 PMC5573342

