# Supplementary material for: Biomarkers in the pathogenesis and diagnosis of Crohn’s perianal fistulas: a systematic review
Source: Tech Coloproctol. 2026 Jun 22;30(1):140. doi: 10.1007/s10151-026-03376-8 (PMC13538245; doi:10.1007/s10151-026-03376-8)
Supplement: Supplementary file 1 [file 10151_2026_3376_MOESM1_ESM.docx]

**Supplementary Material** PubMed and Scopus search strategies used for the systematic review

**Pubmed search strategy:**

(Crohn Enteritis[MeSH Terms] OR “Inflammatory Bowel Diseases”[MeSH Terms] OR “Crohn Disease”[MeSH Terms] OR “Crohn’s Disease” OR “Inflammatory Bowel Disease”)

AND

**((fistula[MeSH Terms] OR “rectal fistula”[MeSH Terms] OR “rectovaginal fistula”[MeSH Terms] OR “vaginal fistula”[MeSH Terms] OR “perianal fistula” OR “perianal Crohn's fistulas” OR “cryptoglandular fistula” OR “anorectal fistula” OR “anovaginal fistula” OR “perirectal fistula” OR “perineal fistula” OR “anal fistula” OR “rectal fistula” OR “rectovaginal fistula” OR “vaginal fistula” OR fistula-in-ano OR fistulising perianal OR fistulizing perianal OR fistulizing perirectal OR “anal suppurations” OR “perianal fistulae” OR “perianal Crohn's fistulae” OR “cryptoglandular fistulae” OR “anorectal fistulae” OR “anovaginal fistulae” OR “perirectal fistulae” OR “perineal fistulae” OR “anal fistulae” OR “rectal fistulae” OR “rectovaginal fistulae” OR “vaginal fistulae” OR fistulae-in-ano) OR ((anal OR rectal OR rectovaginal OR perianal OR “perianal Crohn's” OR cryptoglandular OR anorectal OR anovaginal OR vaginal OR perirectal OR perineal) AND abscess[MeSH Terms]))**

**filters: English, from 2000/1/1 - 2025/05/08 Sort by: Most Recent**

**Scopus search strategy:**

(TITLE-ABS-KEY ((inflammatory AND bowel AND disease) OR ( regional AND (ileitis OR enteritis)) OR (terminal AND ileitis) OR (ileocolitis) OR (granulomatous AND (colitis OR enteritis)) OR (crohn* AND (disease* OR enteritis)))

AND

TITLE-ABS-KEY (((anal OR rectal OR rectovaginal OR perianal OR anorectal OR anovaginal OR vaginal OR perirectal OR perineal OR cryptoglandular) AND (fistula or fistulae OR abcess)) OR (fistula-in-anoor fistulae-in-ano OR (anal AND suppurations) OR ((fistulising OR fistulizing) AND (perianal AND crohn* AND disease)))))

AND

PUBYEAR>1999 AND PUBYEAR<2026 AND (LIMIT-TO (DOCTYPE, "ar") OR LIMIT-TO (DOCTYPE, "re")) AND (LIMIT-TO (LANGUAGE, "English"))
