# Supplementary material for: Biomarkers in the pathogenesis and diagnosis of Crohn’s perianal fistulas: a systematic review
Source: Tech Coloproctol. 2026 Jun 22;30(1):140. doi: 10.1007/s10151-026-03376-8 (PMC13538245; doi:10.1007/s10151-026-03376-8)
Supplement: Supplementary file 2 [file 10151_2026_3376_MOESM2_ESM.docx]

**Supplementary Table 1** Studies Quality assessment

| **Study** | **Tool** | **Study type** | **Selection** | **Comparatibility** | **Outcome** | **Total** |
| --- | --- | --- | --- | --- | --- | --- |
| **PATHOGENESIS STUDIES** |  |  |  |  |  |  |
| **Genetic profile** |  |  |  |  |  |  |
| Vermeire *et al.* [17] | NOS | Case-control | **** | * | *** | 8 |
| Palmieri *et al*. [18] | NOS | Case-control | **** | ** | ** | 8 |
| Jakubowska *et al.* [19] | NOS | Cross-sectional | *** | -- | *** | 6 |
| Henckaerts *et al.* [21] | NOS | Cohort | *** | ** | *** | 8 |
| Latiano *et al*. [22] | NOS | Case-control | **** | * | *** | 8 |
| Freire *et al.* [24] | NOS | Cohort | **** | * | * | 6 |
| Eglinton *et al.* [26] | NOS | Case-control | **** | ** | ** | 8 |
| Connelly *et al.* [25] | NOS | Cohort | **** | ** | ** | 8 |
| Yang *et al.* [27] | NOS | Cohort | **** | ** | ** | 8 |
| Schnitzler *et al.* [29] | NOS | Case-control | *** | ** | *** | 8 |
| Can *et al.* [28] | NOS | Case-control | ** | * | *** | 6 |
| Kaur *et al.* [31] | NOS | Case-control | **** | * | ** | 7 |
| Zhang *et al.* [35] | NOS | Case-control | **** | ** | ** | 8 |
| Cusato *et al.* [41] | NOS | Case-control | **** | ** | ** | 8 |
| Gisbert-Ferrándiz *et al.* [42] | NOS | Case-control | **** | ** | ** | 8 |
| **Microbiological profiles** |  |  |  |  |  |  |
| Tozer *et al.* [30] | NOS | Cross-sectional | ** | -- | *** | 5 |
| Haac *et al.* [32] | NOS | Cross-sectional | **** | -- | *** | 7 |
| Breton *et al.* [38] | NOS | Cross-sectional | ***** | * | *** | 9 |
| **Immunological profiles** |  |  |  |  |  |  |
| Ruffolo *et al*. [20] | NOS | Cross-sectional | ** | -- | *** | 5 |
| Haddow *et al.* [33] | NOS | Cross-sectional | **** | -- | *** | 7 |
| Bruckner *et al.* [37] | **QUIN** | **Translational experimental in vitro** | -- | -- | -- | H/M |
| Rizzo *et al.* [39] | **QUIN** | **Translational experimental in vitro** | -- | -- | -- | H/M |
| Levantovsky *et al****.* [44]** | NOS | Cross-sectional | **** | -- | *** | 7 |
| **Metabonomic Profiles** |  |  |  |  |  |  |
| Adegbola *et al.* [36] | NOS | Cross-sectional | *** | * | *** | 7 |
| **DIAGNOSTIC STUDIES** |  |  |  |  |  |  |
| **Histopathological findings** |  |  |  |  |  |  |
| Ramzan *et al.* [16] | NOS | Cohort | **** | * | *** | 8 |
| Denoya et al. [23] | NOS | Cohort | **** | -- | *** | 7 |
| Han et al. [43] | NOS | Case-control | **** | -- | *** | 7 |
| **Inflammatory profile** |  |  |  |  |  |  |
| Stevens et al. [34] | NOS | Cross-sectional | **** | -- | *** | 7 |
| Becker et al. [40] | NOS | Cross-sectional | **** | * | *** | 8 |
| Perregaard et al. [48] | NOS | Cross-sectional | **** | * | *** | 8 |
| Ma et al. [47] | NOS | Case-control | *** | * | *** | 7 |
| **Immunological profile** |  |  |  |  |  |  |
| Ouboter et al. [45] | NOS | Cross-sectional | ** | -- | *** | 5 |
| **Metabonomic profiles** |  |  |  |  |  |  |
| Zhu et al. [46] | NOS | Case-control | *** | * | *** | 7 |

*H/M* high to moderate, ***NOS*** Newcastle-Ottawa Scale, *QUIN* Quality Assessment Tool for In Vitro Studies
